# The effect of the social and interpersonal-based intervention on calcium consumption among pregnant women

**DOI:** 10.3389/fpubh.2025.1496028

**Published:** 2025-04-14

**Authors:** Neda Heidari, Masoomeh Amiri, Fatemeh Rajati, Behzad Mahaki, Mojgan Rajati

**Affiliations:** ^1^Student Research Committee, Kermanshah University of Medical Sciences, Kermanshah, Iran; ^2^Research Center for Environmental Determinants of Health, Health Institute, Kermanshah University of Medical Sciences, Kermanshah, Iran; ^3^Department of Biostatistics, School of Health, Kermanshah University of Medical Sciences, Kermanshah, Iran; ^4^Department of Obstetrics and Gynecology, School of Medicine, Motazedi Hospital, Kermanshah University of Medical Sciences, Kermanshah, Iran

**Keywords:** behavior change, health Promotion, maternal and infant health, social intervention, personal, calcium intake, women, health education

## Abstract

**Introduction:**

The present study aimed to evaluate the effect of educational intervention based on Pender’s health promotion model on the calcium intake of pregnant women.

**Methods:**

The pregnant women at three to 5 months were recruited using convenient sampling method and randomly assigned either to the control (*n* = 37) or intervention (*n* = 36) groups referred to the health centers in Kermanshah, “Iran,” in 2022, 2023. Participants were randomly assigned to either the intervention group or the control group. The intervention consisted of a series of educational workshops that provided information on the importance of calcium, dietary sources, and practical methods for increasing calcium intake. Participants received personalized dietary plans, and improve knowledge, perceived benefits of action, perceived barriers to action, perceived self-efficacy, activity-related affect, interpersonal influences, situational influences, immediate competing demands and preferences, commitment to plan of action and over 8-week period, and ongoing support through one-month follow-up. The control group received standard prenatal care without additional nutritional education. Pre- and post-intervention assessments measured calcium intake and HPM constructs using validated questionnaires. The food frequency questionnaire was completed before and after the intervention. Data were analyzed in SPSS software version 25.

**Results:**

There is no significant difference between the intervention and control groups at baseline (*p* < 0.05). According to the independent sample *t*-test, all constructs of the Pender’s HPM except for interpersonal influences were significantly improved in the intervention rather than control groups (*p* > 0.05). The repeated measure ANOVA demonstrated a significant difference in the effect of the intervention on the constructs of the knowledge (*F* = 9.40; *p*-value = 0.001), perceived benefits (*F* = 17.24; *p*-value = 0.001), perceived barriers (*F* = 40.80; *p*-value = 0.001), perceived self-efficacy (*F* = 10.90; *p*-value = 0.001), activity-related affect (*F* = 14.85; *p*-value = 0.001), interpersonal influences (*F* = 21.51; *p*-value = 0.001), commitment to a plan of action (*F* = 20.20; *p*-value = 0.001), and immediate competing demands and preferences (*F* = 9.4; *p*-value = 0.001) between the intervention and control groups. The ANOVA demonstrated that the calcium consumption significantly increased in the intervention group (*p* < 0.001).

**Discussion:**

A theory-based educational intervention in the health care system can fill the gap in the successful implementation of nutrition education programs.

## Introduction

Pregnancy as one of the most important periods with commonly medical complications in women’s life, is associated with the enhancement of nutritional needs. Further, pregnancy influences the lifestyle and nutrition behaviors of women and their families (1). The malnutrition or inappropriate nutrition during pregnancy leads to a range of maternal and fetal complications, such as intrauterine growth restriction (IUGR), small for gestational age (SGA) (2), preterm delivery, premature rupture of membranes break, polyhydramnios, oligohydramnios, pre-eclampsia, spontaneous abortions, medically induced labor, fetal death, fetal distress, fetal growth restriction, and malformations (3).

The fetus meets all its needs from the mother during its growth and development and inadequate nutrients in the mother leads to the depletion of the body’s reserves and maternal weight loss (4, 5). The need for calcium in the diet increases during pregnancy and pregnant women, especially in the third trimester are at risk of calcium deficiency (6). Human pregnancy is associated with changes in calcium and bone metabolism, supporting the calcium transport between mother and fetus (7). Milk and dairy products are determinant in providing protein and calcium needed during pregnancy and preventing some complications, such as osteoporosis and tooth decay (8). Studies indicate that consuming more dairy products, such as milk dairy product during pregnancy may reduce the risk of newborn respiratory distress syndrome (9). Calcium deficiencies during pregnancy can lead to retardation of fetal skeletal growth and low level of calcium in the mother’s milk (10). Similarly, women who have experienced multiple births and did not receive adequate calcium intake may be at an increased risk of developing osteoporosis (11). A large number of studies indicated the significant role of calcium in preventing pre-eclampsia (11). Low calcium intake is associated with hypertensive disorders in pregnancy (12). Accordingly, calcium plays significant role in insulin resistant syndrome (13).

World Health Organization (WHO) and Food and Agriculture Organization (FAO) recommended dietary calcium intake of 1,200 mg/day for pregnant women (14). Some studies demonstrated that the calcium intake among Asian women is lower than the recommended values (15, 16) and the prevalence of calcium deficiency in different months of pregnancy varies from 19 to 33.5% among Iranian mothers (17, 18). A wide number of foods contain calcium, such as milk and milk products (providing 34–60% of calcium intake amongst adults) (19) and cereals and cereal products (providing 30% of calcium intake) (20). Milk and milk products are recognized as the main source of high bioavailable calcium with an average calcium content of 1,150 mg/L (21).

Research has shown that in various health-related behaviors such as nutritional behavior, interpersonal and certain social factors can play a pivotal role (22). While the World Health Organization has established nutritional and health guidelines for pregnant women these guidelines are implemented through healthcare systems, many of them fail to adequately account for interpersonal and social determinants of nutritional behaviors (23). Specifically, with regard to the nutrition of pregnant women, these guidelines have struggled to effectively improve the nutritional behaviors of this population, particularly in developing countries (24). Therefore, behavioral intervention about increased dairy product consumption seem to meet the extra needs of pregnant women. Recommendation for consuming calcium-rich foods includes a part of the routine health care of pregnant women in Iran (18). However, reaching sufficient amounts of calcium is still a challengeable issue among Iranian pregnant women (25).

Studies indicated that desirable nutritional behaviors can be promoted by improving self-efficacy, commitment to action, positive emotions related to behavior, the perceived benefits of performing the behavior, perceived sensitivity, perceived severity, and the reduction of perceived barriers among obese and overweight women (26). Pender’s health promotion model, including many of the afore-mentioned variables (27), can be effective for intervention among pregnant women. Based on the available evidence about the effectiveness of Pender’s health promotion model in nutritional behaviors, the model seems to be successfully used in improving calcium consumption behavior of pregnant women (26, 28). Therefore, the present study aimed to the effect of the social and interpersonal-based intervention on calcium consumption among pregnant women.

## Methods

### Study design and participants

This was a semi-experimental study conducted in Kermanshah, from November 2022 to January 2023. Pregnant women referred to three health centers with similar socioeconomic, social, and cultural status were selected using convenient sampling and randomly assigned to control (*n* = 37) or intervention (*n* = 36) groups.

The inclusion criteria were pregnant women with gestational age of three to 5 months and literacy. Exclusion criteria included mothers with acute digestive disorders, such as absorption disorders and irritable bowel syndrome, as well as pregnancy complications like eclampsia and pre-eclampsia, as diagnosed by the center doctor, parity considerations, use of medications such as corticosteroid impacting calcium metabolism, mental health issues affecting dietary habits, and variations in social support.

**Figure 1:**
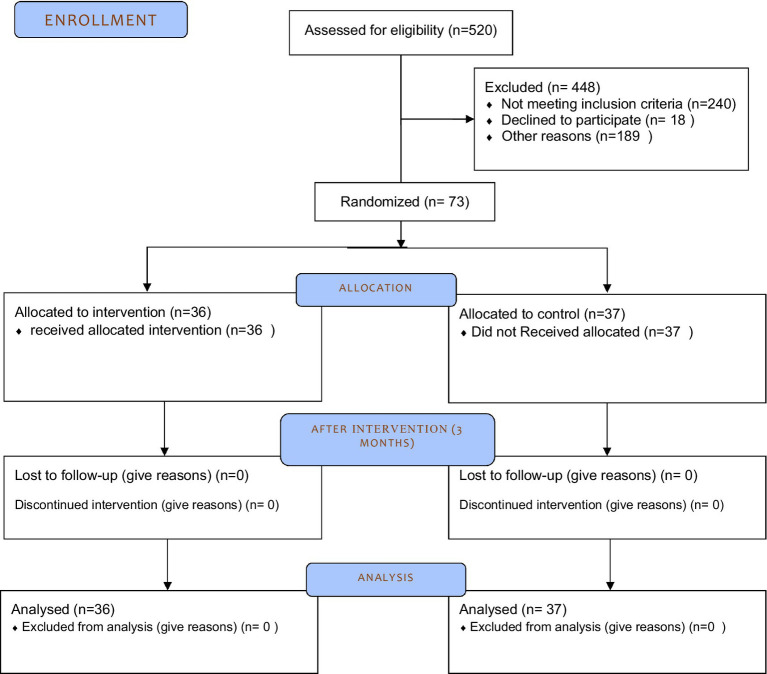
Flow diagram of recruitment of study participants.

From all eligible pregnant women, 73 individuals were recruited in the study considering the inclusion and exclusion criteria (Figure 1).

Before the intervention, all participants provided informed consent after being explained the study objectives. They were assured of confidentiality and their right to withdraw from the study at any time.

### Measurements

#### Demographic characteristics questionnaire

A researcher-made questionnaire collected basic demographic information, including age, education level, occupation, family income, residential area, and type of health insurance.

#### Nutritional behavior questionnaire based on Pender’s health promotion model

This 60-item questionnaire measured various constructs of Pender’s model on a 5-point Likert scale based on the previous studies verified and used (26). It assessed participants’ knowledge, perceived benefits and barriers, self-efficacy, activity-related affect, situational and interpersonal influences, commitment to the plan of action, and immediate competing demands and preferences.

The questionnaire items were developed based on a literature review and validated through content and face validity assessments by 14 experts. The average content validity ratio (CVR) was 0.82, and the average content validity index (CVI) was 0.8, indicating good validity. The reliability of the questionnaire was confirmed using a test–retest method with a 5-day interval among 24 pregnant women, resulting in an Interclass Correlation Coefficient (ICC) of 0.73 and a Cronbach’s alpha of 0.87.

#### Calcium intake assessment

Participants completed non-consecutive three-day food records (3D-FRs) to evaluate their calcium dietary intake at the beginning and end of the intervention (29). They were instructed to record everything they consumed during 3 days, with two normal days and 1 day off. The consumed raw and cooked ingredients were converted to grams or milliliters, and the calcium content was analyzed using the Nutritionist IV software and the United States Department of Agriculture (USDA) food table, which was modified for Iranian foods (30).

#### Procedure

The intervention lasted 8 weeks and comprised three training sessions utilizing a lecture-based approach, addressing Bloom’s three cognitive, emotional, and psychomotor domains. Additionally, three sessions employed telephone discussions, counseling, role-playing, and feedback on program implementation to enhance each construct. Each session lasted 90 min.

During the intervention, pregnant women received follow-up via SMS, which included practical instructions for implementing calcium-rich dietary sources and utilizing muscle relaxation and meditation clips at home and outside the health center. In the subsequent sessions, participants shared their challenges—such as selecting calcium-rich foods over unhealthy options—and their successes with both peers and study researchers. Teaching aids for each session included video projectors and PowerPoint slides. The educational content was designed and delivered through educational clips, posters, and pamphlets tailored for pregnant mothers. After 8 weeks, the same intervention was conducted for control group.

##### Knowledge

The intervention utilized a multi-pronged approach to improve women’s knowledge about calcium needs and calcium-rich foods. This included providing:

a) Three educational sessions
b) Informational pamphlets and educational videos
c) Group discussions on calcium needs during pregnancy and breastfeeding
d) Assignments for participants to search the web and find scientific information on calcium-rich foods

##### Perceived benefits

The intervention highlighted the important structural, metabolic, and regulatory roles that calcium plays in the body. Specific benefits discussed included the prevention of preeclampsia, preterm birth, low birth weight, and osteoporosis.

##### Perceived barriers

To address perceived barriers, the intervention focused on:

a) Methods for replacing ingredients and the proper ways of consuming dairy products
b) The best and worst times for milk consumption
c) Providing a range of dairy substitutes like almond milk, coconut milk, soy milk, beans, fish, fruits, and vegetables

##### Self-efficacy

Several strategies were used to improve self-efficacy for calcium-rich food consumption:

a) Mastery experiences through gradual behavior changes and step-by-step implementation
b) Role modeling from women who overcame milk intolerance and barriers
c) Verbal persuasion, feedback, and encouragement from the investigator
d) Training in relaxation techniques to manage stress and tensions

##### Activity-related affects

The intervention introduced a variety of calcium-rich product replacements and aimed to address any fears or negative feelings about not following the recommended diet.

##### Interpersonal influencers

Since access to peers was limited, the intervention focused on involving spouses. Spouses were invited to an educational session, provided brochures, and asked to encourage the mothers and avoid purchasing competing foods.

##### Competing influencers

The participants discussed ingredients or factors (e.g., soft drinks) that interfered with dairy and milk consumption, and collaborated to find solutions.

##### Commitment to a plan of action

The intervention addressed the participants’ commitment and emphasized the importance of continuing the target calcium-rich food consumption behavior.

##### Immediate competing demands and preferences

The intervention explored the women’s desires for items like desserts and soft drinks that competed with milk and dairy product consumption, and discussed ways to manage these competing demands.

### Statistical analysis

The outlier variables were removed using SPSS software, Ver. 22 by Explore command. None of the variables related to Pender’s constructs had outliers. The difference of the amount of calcium intake before and after the intervention in three participants had outlier in the intervention group and were excluded from the analysis. The difference of the amount of calcium intake before and after the intervention had no outlier in the control group.

We began by calculating descriptive statistics for all key variables, including means, standard deviations, and frequencies, to summarize participant characteristics and baseline measurements.

Standard Error of the Skewness and Kurtosis (between +2 and −2 values) were used to evaluate the normality of quantitative variables. After confirming the normality of the data, the independent sample *t*-test was used to measure the significant difference of normal quantitative variables, including income, gestational age, systolic blood pressure, and diastolic blood pressure, at the baseline. The Chi-square test was applied to assess the homogeneity of the classified quantitative variables, including age, parity, number of children, and nominal variables, including education, job, spouse’s education, and comorbidity between the control and intervention groups at baseline. The Mauchly’s test of sphericity showed that the assumption of sphericity of the data is not met (*p*-value<0.001).

Since the outcome variables included knowledge, perceived benefits, perceived barriers, perceived self-efficacy, activity-related affect, situational influences, interpersonal influences, situational influences, and commitment to a plan of action were measured after the intervention and 1 month after the intervention (follow up), repeated measure ANOVA test was employed to measure the effect of the intervention over time. First, the effect of the time was assessed independent of the effect of time. Then, the effect of groups was assessed independent of the effect of time. The final result of the test was evaluated in the form of interaction time × group.

Mauchly’s test of sphericity validates the repeated measure ANOVA, which was performed before the test. If the significance level is less than 0.05, H_0_ is rejected and H_1_ is confirmed. If H_0_ is rejected, the sphericity of the variance–covariance matrix of the dependent variable should be accepted and one of the Greenhouse Geisser, Haven-Flat, or lower limit tests should be used. These tests correct the degree of freedom and if H_0_ of Mauchly’s test is rejected, Greenhouse Geisser test is used in the present study.

Given that calcium consumption was measured only before and after the intervention and there was a significant difference in the amount of calcium intake at the baseline between the control and intervention groups, to control for baseline differences between the intervention and control groups, we employed Analysis of Covariance (ANCOVA). This method allowed us to adjust for pre-existing differences in calcium intake and other relevant demographic variables, ensuring that the observed effects of the intervention were not confounded by these baseline characteristics. Prior to conducting ANCOVA, we assessed the assumptions of normality and homogeneity of variances.

To enhance clarity and understanding, we included visualizations comprising charts to depict the changes in calcium intake and the constructs of HPM in each group over time. The statistical significance level was considered as *p*-value <0.05.

## Results

The mean age of participants in the control group was higher than that in the intervention group, with values of 27.19 ± 6.3 years and 30.77 ± 5.7 years, respectively; however, this difference was not statistically significant. Similarly, the mean gestational age was slightly greater in the control group (16.8 ± 3.2 weeks) compared to the intervention group (16.4 ± 3.7 weeks), but this difference was also not significant. In terms of blood pressure, the mean systolic blood pressure was 11.6 ± 0.8 in the control group, while it was 11.2 ± 9.0 in the intervention group, with no significant difference noted.

The distribution of variables was normal. There was no significant difference in income, gestational age, systolic and diastolic blood pressure at baseline between the groups. The difference in systolic blood pressure had no clinical value. There was no statistically significant difference between the groups at baseline (Table 1).

**Table 1:** The comparison of quantitative variables in the intervention and control groups at baseline.

Variables	Control	Intervention	*T*-value	*P*-value
	SD ± Mean	SD ± mean		
Calcium intake amount	185.88 ± 580.122	398.591 ± 732.15	2.016	0.05
Gestational age (week)	2.3 ± 8.16	3.7 ± 16.4	436	664
Income (toman)	09.1 ± 1.3	02.1 ± 6.2	526	71
Systolic blood pressure (mm Hg)	8.0 ± 6.11	9.0 ± 2.11	9.1	57
Diastolic blood pressure (mm Hg)	7.0 ± 6.7	8.0 ± 2.7	9.1	50

The Chi-square test indicated no significant differences in categorical demographic variables between the intervention and control groups at baseline. This includes age (≥28 years and < 28 years), education levels (illiterate: 33.3%; diploma and under diploma: 66.6%; academic education: 33.3%), employment status (employed vs. unemployed), spouse’s education, number of children, parity, and comorbidities (hypertension, diabetes, and cancer) (*p* < 0.05) (Table 2).

**Table 2:** The demographic variables of pregnant women in the intervention and control groups at baseline.

Variable / Group		Control n (%)	Intervention n (%)	*P-*value*
Age	Age 28 >	16 (44.4)	20 (55.6)	0.346
	Age 28 ≤	20 (55.6)	16 (44.4)	
Education	High school	10 (41.7)	14 (58.3)	0.075
	Diploma	16 (69.9)	7 (30.4)	
	Bachelor degree and higher	(40)10	(60)15	
Job	Employed	10 (62.5)	6 (37.5)	0.257
	Un-Employed	26 (46.4)	30 (53.6)	
Spouse education	High school	(9/52)9	(1/47)8	739/0
	Diploma	(9/52)18	(1/47)16	
	Bachelor degree and higher	(9/42)9	(1/57)12	
Parity	1	(1/32)9	(9/67)19	076/0
	2	(5/64)20	(5/35)11	
	3	(8/53)7	(2/46)6	
Number of children	1	(3/39)11	(7/60)17	102/0
	2	(6/63)21	(4/36)12	
	3	(4/36)4	(6/63)7	
Comorbidity	No	(50)32	(50)32	645/0
	Yes	(4)50	(4)50	

The independent sample t-test showed significant changes in the mean scores of knowledge (*p* < 0.001, *t* = 4.20), perceived benefits (*p* < 0.001, *t* = 6.26), barriers (*p* < 0.001, *t* = 6.87), self-efficacy (*p* < 0.001, *t* = 3.56), activity-related affect (*p* < 0.001, *t* = 4.70), situational influences (*p* < 0.001, *t* = 3.19), commitment (*p* = 0.001, *t* = 4.08), and immediate competing demands (*p* < 0.001, *t* = −3.99) before and after the intervention, except for spouse’s social support (*p* < 0.20, *t* = 1.28).

Muchly’s test showed the variance–covariance matrix was not spherical for most variables (*p* > 0.05), so the Greenhouse–Geisser correction was used to correct the degrees of freedom.

The results of the repeated measure ANOVA test at baseline indicated that the trend of changes independent of the effect of the two groups in the mean scores of knowledge (*p*-value = 0.001; *F* = 7.7), perceived benefits (*p*-value <0.001; *F* = 38.2), perceived self-efficacy (*p*-value <0.002; *F* = 6.4), activity-related affect (*p*-value <0.001; *F* = 25.08), interpersonal influences (*p*-value =0.043; *F* = 3.21), situational influences (*p*-value <0.001; *F* = 12.82), commitment to a plan of action (*p*-value <0.001; *F* = 25.43), and the construct immediate competing demands and preferences (*p*-value <0.001; *t* = −3.99) were incremental and significant from baseline to after the intervention over time (Table 3).

**Table 3:** The changes in the variables related to the Pender’s HPM before and after the intervention and during the follow-up in the control and intervention groups.

Variable		Baseline	After intervention	Follow up (one month)	Time effect	Group effect	F**	*P*-value**
		Mean ± SD	Mean ± SD	Mean ± SD				
Knowledge	Intervention	2.6 ± 0.89	4.08 ± 1.3	4.02 ± 1.4	<0.001	0.003	9.4^a^	0.001
	Control	2.4 ± 1.05	2.3 ± 1.5	2.5 ± 1.6				
*P*-value*		0.473	0.001	0.001				
Perceived benefits	Intervention	19.5 ± 1.5	22.08 ± 1.7	22.3 ± 2.08	<0.001	0.001	17.24^b^	0.001
	Control	19.5 ± 1.6	19.7 ± 1.4	20.4 ± 2.1				
*P*-value*		0.94	0.001	0.001				
Perceived barriers	Intervention	9.4 ± 1.2	6.8 ± 1.3	6.7 ± 1.3	<0.001	0.001	40.8^a^	0.001
	Control	9.5 ± 1.4	9.6 ± 1.1	9.5 ± 1.2				
*P*-value*		0.791	0.001	0.001				
Perceived self-efficacy	Intervention	13.2 ± 2.04	16.9 ± 4.9	16.02 ± 4.4	0.001<	0.005	10.90^a^	0.001
	Control	13.5 ± 4.1	13.08 ± 2.01	13.05 ± 2.2				
*P*-value*		0.718	0.001	0.001				
Activity-related affect	Intervention	16.1 ± 4.4	18.3 ± 1.7	17.9 ± 1.7	<0.001	0.001	14.85^a^	0.001
	Control	15.5 ± 1.5	15.8 ± 1.2	15.6 ± 1.2				
*P*-value*		0.059	0.001	0.001				
Interpersonal influences	Intervention	38.6 ± 9.1	43 ± 8.2	42.2 ± 8.6	0.068	0.002	1.2^a^	0.296
	Control	35.5 ± 6.09	36.5 ± 9.8	36.3 ± 9.9				
*P*-value*		0.171	0.001	0.001				
Situational influences	Intervention	29.05 ± 4.3	33.1 ± 3.5	32 ± 3.5	<0.001	0.001	21.51^a^	0.001
	Control	27.9 ± 2.3	28.4 ± 5.7	26.9 ± 2.7				
*P*-value*		0.089	0.004	0.01				
Commitment to plan	Intervention	24 ± 3.9	29.1 ± 3.8	30.6 ± 5.3	0.001	0.001	20.2^a^	0.001
	Control	25.2 ± 3.5	25.8 ± 2.2	25.5 ± 1.9				
*P*-value*		0.154	0.001	0.001				
Immediate competing demands preferences	Intervention	3.80 ± 0.3	5.30 ± 0.18	4.9 ± 0.19	<0.001	<0.001	12.82^a^	<0.001
	Control	3.63 ± 0.22	3.69 ± 0.22	3.58 ± 0.22				
*P*-value*		0.14	0.001	0.001				

*Independent sample t-test between intervention and control. **Tests of Within-Subjects Effects (interaction Time * group).

a Greenhouse–Geisser correction.

b Sphericity Assumed.

The repeated measure ANOVA also showed the studied variables were significantly different between intervention and control groups, regardless of time. Knowledge (*p* < 0.001, *F* = 17.15), perceived benefits (*p* < 0.001, *F* = 27.32), barriers (*p* < 0.001, *F* = 67.39), self-efficacy (*p* = 0.001, *F* = 11.97), activity-related affect (*p* < 0.001, *F* = 41.78), situational influences (*p* < 0.001, *F* = 24.96), interpersonal influences (*p* = 0.002, *F* = 10.21), commitment (*p* < 0.001, *F* = 14.32), and immediate competing demands (*p* < 0.001, *F* = 16.26) were significantly improved.

The interaction time × group effect was significant for all constructs of Pender’s HPM except for interpersonal influences construct. Table 3 illustrates the F- and *p*-value for the tests used for all constructs of Pender’s HPM.

**Figure 2:**
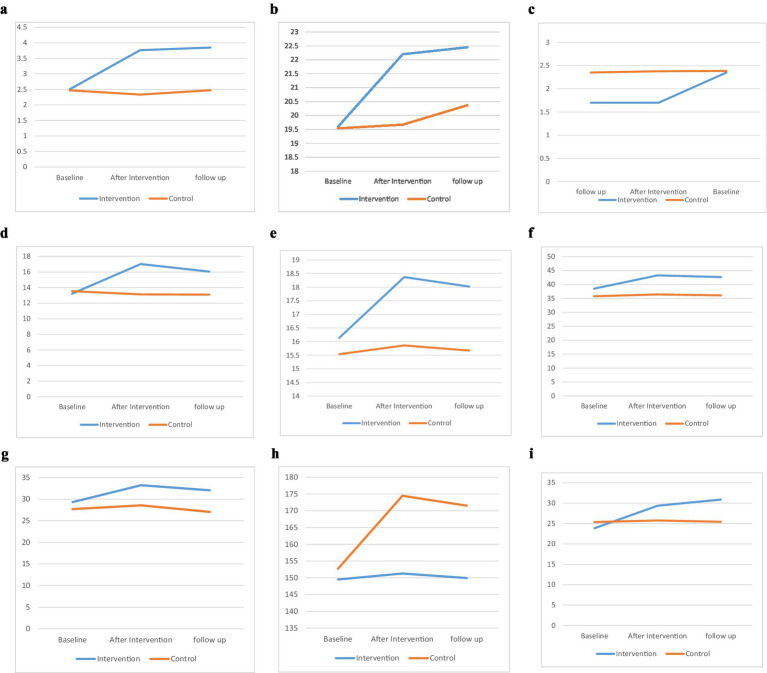
The effect of the intervention on Pender’s HPM on knowledege **(A)** perceived benefits of action **(b)**, perceived barriers to action **(c)**, perceived self-efficacy **(d)**, activity-related affect **(e)**, interpersonal influences **(f)**, situational influences **(g)** immediate competing demands and preferences **(h)**, commitment to plan of action **(i)** in control and intervention groups.

Figure 2 displays the trend of changes in the mean score of constructs of Pender’s HPM in baseline, after intervention, and follow-up according to the control and intervention groups.

ANCOVA was used with adjustment of pre-test role to determine the effect of educational intervention based on Pender’s HPM on avarage calcium intake. The results showed that the intervention was effective and there is a significant difference in calcium intake (mg/day) between two groups (*p* < 0.001). (Table 4).

**Table 4:** The comparison of average calcium intake (mg/day) in the intervention and control groups.

Group	Baseline calcium	Calcium after intervention	F	*P*-value
	Mean ± SD	Mean ± SD		
Intervention	398.59 ± 732.15	422.50 ± 890.280	12.1	0.001
Control	188.60 ± 580.12	224.74 ± 620.10		

## Discussion

This study provides significant insights into the effectiveness of Pender’s Health Promotion Model (HPM) in enhancing calcium consumption among pregnant women, a demographic often overlooked in nutritional interventions. The educational intervention based on Pender’s Health Promotion Model effectively improved the model’s constructs, except for social support, and increased the calcium intake of pregnant women. The intervention significantly enhanced the women’s knowledge, perceived benefits, perceived barriers, perceived self-efficacy, activity-related affect, situational influences, commitment to a plan of action, and reduced immediate competing demands and preferences, both immediately after and 1 month later. Unlike previous studies that primarily focused on general dietary behaviors, this research specifically targets calcium intake, addressing a critical nutritional gap during pregnancy. The incorporation of social and interpersonal factors into the intervention design is particularly noteworthy, as it emphasizes the importance of contextual influences on dietary choices. This approach not only enriches the existing literature but also suggests that tailored educational strategies can effectively modify health behaviors in culturally specific settings, such as Iran.

Previous studies have also found that increasing perceived benefits and reducing perceived barriers can lead to improved self-efficacy in health behaviors, consistent with the findings of the present study (31). When compared to similar studies, our findings align with those of Vahedian Shahroodi et al. (32), who demonstrated that enhancing perceived benefits and reducing barriers significantly improved health behaviors among women. However, this study extends those findings by explicitly linking the constructs of Pender’s HPM to increased calcium intake, providing a more detailed understanding of how these constructs interact within the context of pregnancy. Furthermore, while many studies have reported improvements in general nutritional behaviors, our research uniquely quantifies the impact of a theory-based intervention on specific dietary outcomes, such as calcium consumption. This distinction highlights the potential for Pender’s HPM to serve as a robust framework for future interventions aimed at improving maternal nutrition, thereby contributing to better health outcomes for both mothers and their infants. Removing perceived barriers are an important factor in the process of self-care to perform healthy behaviors (33).

The self-efficacy of calcium consumption was improved through Bandura’s key strategies including mastery experiences, social modeling, verbal persuasion, and psychological responses (34). Participants were guided through step-by-step dietary changes, engaging successful pregnant women as role models, and receiving encouragement and feedback. Relaxation techniques addressed stress related to the behavior change. This multifaceted approach aligns with Bandura’s recommendations and is consistent with previous research (35). Vahedian Shahroodi et al. (32) similarly employed strategies such as lectures and self-monitoring to improve self-efficacy, while Wise et al. (36) found that involving pregnant women in healthy food preparation motivated their consumption. Studies have consistently demonstrated the predictive role of self-efficacy in shaping nutritional behaviors (37, 38).

The present study found that the activity-related affect score, reflecting feelings about performing the behavior, significantly increased in the intervention group after the intervention and during follow-up compared to the control group. This is significant, as Pender’s model suggests activity-related affect can explain 58% of the commitment to the behavior (39). Similarly, Goodarzi-Khoigani et al. (28) found a positive relationship between dairy consumption and Pender’s model constructs, which improved with educational intervention. The intervention addressed the negative feelings some mothers had towards milk consumption due to digestive issues, resolving their fear of potential consequences like vomiting, and thus improving their activity-related affect.

Interpersonal influences are defined as cognitions concerning the behaviors, beliefs, or attitudes that decide individuals’ predisposition to involve in healthy behaviors (40).

Interpersonal influences, such as social support from spouses, had limited impact on healthy behaviors after the intervention. The focus on emotional support was insufficient, and other forms of spousal support were less investigated. Cognitive and physiological factors may be more influential than spousal encouragement in adopting behaviors like consuming calcium-rich foods, due to issues like vomiting and taste preferences. The present study was in line with the study of Goodarzi-Khoigani et al. (28)in the field of interpersonal influences in performing the desired behavior.

Perceived barriers and situational influences were predictive of commitment to a plan of action. Consistent with the findings of Elseifi et al. (41) on breakfast consumption among students. The present study revealed that educational interventions, aimed at not consuming competitors and raising mothers’ knowledge of tempting situations to consume foods competing with calcium intake, can be effective until the follow-up period. However, some studies demonstrated no significant relationship between situational influences and oral health-related behaviors (42).

Commitment to the plan of action showed an increasing trend after the intervention and during follow-up. Previous studies demonstrated that performing the related behavior significantly improves among pregnant women with greater commitment to the use of supplements. Based on the results of the early studies, the greater the commitment of pregnant mothers to the use of supplements, the more significant the associated behavior (43).

Commitment, driven by an internal locus of control, can motivate individuals to adopt healthy behaviors despite obstacles (44). In the present study, the interventions could create an internal locus of control for individuals and increase their commitment to the implementation of the instruction.

Existing research has demonstrated that the desire to consume calcium-rich foods often competes with the intake of soft drinks, jam, ice cream, and other energy-dense, nutrient-poor foods (45). Previous studies have shown that interventions based on Pender’s Health Promotion Model (HPM) have been effective in addressing the role of immediate competing demands (46), although the study by Dehdari et al. (47) reported no significant change in this construct following the implementation of the intervention. Effective health promotion programs should focus on utilizing motivational strategies across various healthcare settings (48).

In our study, pregnant women were recommended to use calcium supplements and to take Vitamin D if they had a deficiency. Therefore, the values of calcium reported are solely related to food sources. Despite the intervention leading to an improvement of about 160 mg per day, the mean calcium intake remained below the recommended 1,200 mg/day for pregnant women. This confirms that supplementation is still necessary for pregnant women.

The use of nutritional outcomes, in addition to questionnaire data, provided an opportunity to evaluate the intervention’s impact in a clinical setting. Randomization in the allocation of participants to intervention and control groups improved the ability to control for confounding factors. All interventions were implemented according to the constructs of Pender’s Health Promotion Model.

### Limitation

Our study had some limitations including.

a) *Self-Report Bias*: Relying on self-reported data can introduce bias, as participants may overestimate their calcium intake or misreport their dietary habits. To mitigate this, we emphasized the importance of using validated food frequency questionnaires.
b) *Sampling Constraints*: The convenience sampling method employed in our study may limit the generalizability of our findings.
c) *Focus on Dairy Intake*: Our study concentrated specifically on dairy products as a primary source of calcium. While this focus is relevant, we acknowledge that other calcium sources, such as leafy greens and fortified foods, were not included.
d) *Limited Interpersonal Influences:* As noted, the impact of interpersonal influences, particularly spousal support, was limited in our results, suggesting that future interventions should consider a broader range of social support mechanisms, including peer support groups or community engagement strategies.
e) *Short Follow-Up Period:* The one-month follow-up may not fully capture the long-term effects of the intervention on calcium intake. We recommend extending follow-up periods in future research to better assess the sustainability of behavior change over time.

## Conclusion

Based on the results, Pender’s HPM can effectively improve the consumption behavior of foods containing calcium, such as milk and dairy products among pregnant women. All constructs of Pender’s HPM, except for interpersonal influencers, showed a significant increase after the intervention and in a one-month follow-up period. The amount of calcium intake of mothers increased after the three-month intervention period by improving Pender’s HBM constructs. Pregnant women should be empowered to make healthy behaviors, and appropriate health education interventions should be developed. Testing HPM is needed to support its predictive power in dairy behaviors. It also is suggested that health care providers use Pender’s HPM for other health-related behaviors.

### Implications for practice and research

The findings highlight the potential of educational interventions based on Pender’s Health Promotion Model to promote calcium intake among pregnant women by targeting key determinants. The model emphasizes the importance of tailoring educational interventions to meet the specific needs and cultural contexts of target populations. Future interventions in different settings, such as rural or underserved urban areas, could adapt the components of HPM to address local dietary habits, beliefs, and barriers to calcium consumption. This customization can enhance participant engagement and improve health needs in high risk population such as children and older adult.

Healthcare providers should integrate these tailored strategies into prenatal care to empower pregnant women and address maternal nutrition challenges. Future research should explore applying health promotion models to other populations and behaviors to develop targeted interventions and enhance public health initiatives.

### Implications for policy

The results highlight the value of incorporating a health promotion model into the development and implementation of nutritional guidelines and interventions within the healthcare system. This theory-driven approach offers a comprehensive framework to promote healthy dietary behaviors among pregnant women by addressing multidimensional factors. Healthcare providers should consider adopting Pender’s model as a guiding principle when designing, delivering, and evaluating nutrition education programs for pregnant women, empowering them to overcome barriers, enhance self-efficacy, and sustain calcium-rich food consumption habits that support optimal maternal and fetal outcomes.

## Data Availability

The raw data supporting the conclusions of this article will be made available by the authors, without undue reservation.
