# Supplementary material for: The effect of the social and interpersonal-based intervention on calcium consumption among pregnant women
Source: Front Public Health. 2025 Apr 14;13:1496028. doi: 10.3389/fpubh.2025.1496028 (PMC12034691; doi:10.3389/fpubh.2025.1496028)
Supplement: Supplementary file 1 [file Table_1.docx]

**Demographic Characteristics**

| **Question** | **Answer** |
| --- | --- |
| 1. Age: | ............. |
| 2. Education: |  |
| 3. Job: |  |
| 4. Spouse’s Education: |  |
| 5. What number parity is this? |  |
| 6. What number child is this? |  |
| 7. Income level (in million Toman): |  |
| 8. How many weeks pregnant are you? |  |
| 9. Recorded underlying diseases: |  |
| 10. Last recorded blood pressure: |  |

**Knowledge**

*Please read each question carefully and select only one option:*

| **Question** | **True** | **False** |
| --- | --- | --- |
| 1. If I do not consume enough milk and dairy products, I will develop tooth decay in the future. |  |  |
| 2. If I do not consume milk and dairy products, my fetus will never suffer from calcium deficiency. |  |  |
| 3. During pregnancy, my body's need for calcium increases. |  |  |
| 4. Preventing osteoporosis is one of the benefits of consuming milk and dairy products during pregnancy. |  |  |
| 5. Consuming milk and dairy products during pregnancy helps in the formation of the fetus's bones, heart, nerves, and joints. |  |  |
| 6. The best time to consume milk and dairy products is the night before sleep. |  |  |
| 7. Drinking orange juice along with milk and dairy products helps in their absorption. |  |  |
| 8. One of the side effects of excessive consumption of milk and dairy products is bloating. |  |  |

**Perceived Benefits of action**

*Please read each sentence carefully and select only one option:*

| **Question** | **Strongly Agree** | **Agree** | **Disagree** | **Strongly Disagree** |
| --- | --- | --- | --- | --- |
| 1. I believe that adhering to the consumption of milk and dairy products during pregnancy is essential for my health and my baby's. |  |  |  |  |
| 2. I find it easy to follow a diet during pregnancy. |  |  |  |  |
| 3. I believe that adequate consumption of milk and dairy products during pregnancy can prevent tooth decay. |  |  |  |  |
| 4. I believe that following the recommended diet does not incur significant financial costs. |  |  |  |  |
| 5. I think that with the start of pregnancy, I should increase my consumption of milk and dairy products. |  |  |  |  |

**Perceived Barriers to Action**

*Please read each sentence carefully and select only one option:*

| **Question** | **Strongly Agree** | **Agree** | **Disagree** | **Strongly Disagree** |
| --- | --- | --- | --- | --- |
| 1. Due to the busyness of life, I cannot consume the daily required amount of milk and dairy products. |  |  |  |  |
| 2. Consuming milk and dairy products during pregnancy causes digestive problems. |  |  |  |  |
| 3. Following a diet that includes milk and dairy products makes me feel tired. |  |  |  |  |
| 4. The cost of obtaining milk and dairy products prevents me from consuming them regularly. |  |  |  |  |

**Perceived Self-Efficacy**

*Please indicate your level of confidence from 1 to 5 (1 being the least confidence and 5 the most):*

| **Question** | **1** | **2** | **3** | **4** | **5** |
| --- | --- | --- | --- | --- | --- |
| 1. How confident are you that you can consume enough milk and dairy products? |  |  |  |  |  |
| 2. How capable are you of following a milk and dairy diet even at parties? |  |  |  |  |  |
| 3. How capable are you of increasing your consumption of milk and dairy products in your diet? |  |  |  |  |  |
| 4. How capable are you of ensuring the required calcium intake during pregnancy regardless of your economic situation? |  |  |  |  |  |
| 5. How capable are you of consuming milk and dairy products despite digestive issues? |  |  |  |  |  |

**Activity-related Affects**

*Please read each sentence carefully and select only one option:*

| **Question** | **Strongly Agree** | **Agree** | **Disagree** | **Strongly Disagree** |
| --- | --- | --- | --- | --- |
| 1. Following the recommended diet is not boring for me. |  |  |  |  |
| 2. I fear the consequences of not following the diet. |  |  |  |  |
| 3. I feel satisfied with my current dietary style. |  |  |  |  |
| 4. I feel happy following my current diet. |  |  |  |  |
| 5. My life circumstances are very good due to my current diet. |  |  |  |  |

**Situational Influencers**

*Please read each sentence carefully and select only one option:*

| **Question** | **Always** | **Often** | **Sometimes** | **Rarely** | **Never** |
| --- | --- | --- | --- | --- | --- |
| 1. My pregnancy makes me pay attention to my diet. |  |  |  |  |  |
| 2. Recommendations from my doctor make me pay attention to my diet. |  |  |  |  |  |
| 3. Seeing elderly individuals with bone issues makes me pay attention to my nutrition. |  |  |  |  |  |
| 4. My spouse's conversations make me pay attention to my diet. |  |  |  |  |  |
| 5. When I receive results from my tests, I pay attention to my diet. |  |  |  |  |  |
| 6. When I have guests, I pay attention to my diet. |  |  |  |  |  |
| 7. Reading scientific materials about nutrition during pregnancy compels me to follow a healthy diet. |  |  |  |  |  |
| 8. Fear for the future and my baby's health makes me pay more attention to my nutrition. |  |  |  |  |  |

**Spousal Social Support**

*Please indicate your level of confidence from 1 to 5 (1 being the least confidence and 5 the most):*

| **Question** | **1** | **2** | **3** | **4** | **5** |
| --- | --- | --- | --- | --- | --- |
| 1. How much do you feel at ease when you talk to him/her? |  |  |  |  |  |
| 2. How well does he/she understand your situation? |  |  |  |  |  |
| 3. How much does he/she willingly listen to what you say? |  |  |  |  |  |
| 4. How concerned is he/she about your well-being? |  |  |  |  |  |
| 5. How much do you rely on him/her to solve your problems? |  |  |  |  |  |
| 6. How effective is he/she in reducing your stress? |  |  |  |  |  |
| 7. How much attention does he/she pay to your diet? |  |  |  |  |  |
| 8. How much time does he/she spend ensuring you have access to appropriate food? |  |  |  |  |  |
| 9. How much support does he/she provide for managing your health issues? |  |  |  |  |  |
| 10. How much does he/she assist you in fulfilling your responsibilities? |  |  |  |  |  |
| 11. How much does he/she monitor your diet? |  |  |  |  |  |

**Commitment to Plan of Action**

*How would you describe your situation regarding each of the following options?*

| **Question** | **Always** | **Often** | **Sometimes** | **Rarely** | **Never** |
| --- | --- | --- | --- | --- | --- |
| 1. How committed do you feel to consuming milk throughout the day? |  |  |  |  |  |
| 2. How important is it for you to follow the recommended diet during pregnancy? |  |  |  |  |  |
| 3. How committed are you to ensuring that life's busyness does not hinder your diet? |  |  |  |  |  |
| 4. How committed are you to consuming nutritious and calcium-rich foods over tasty ones at parties? |  |  |  |  |  |
| 5. How committed are you to obtaining and consuming the necessary milk and dairy products during pregnancy regardless of economic conditions? |  |  |  |  |  |
| 6. How committed do you feel to using appropriate substitutes if you cannot obtain your daily milk and dairy products? |  |  |  |  |  |

**Immediate Competing Demands and Preferences**

*Which of the following options are you personally inclined to choose? (These questions focus solely on your personal preferences for food choices.)*

1. Yoghurt or Soda?
2. Deleter or Yoghurt?
3. Full-fat or Low-fat dairy products?
4. Sweets or Ice Cream?
5. Milk or Syrup?
6. Yogurt or Salad?
7. Butter and Jam or Cheese?
